# Supplementary material for: Pathogenicity of Beauveria bassiana PfBb and Immune Responses of a Non-Target Host, Spodoptera frugiperda (Lepidoptera: Noctuidae)
Source: Insects. 2022 Oct 8;13(10):914. doi: 10.3390/insects13100914 (PMC9604019; doi:10.3390/insects13100914)
Supplement: Supplementary file 1 [file insects-13-00914-s001.zip › insects-1924726-supplementary.pdf]

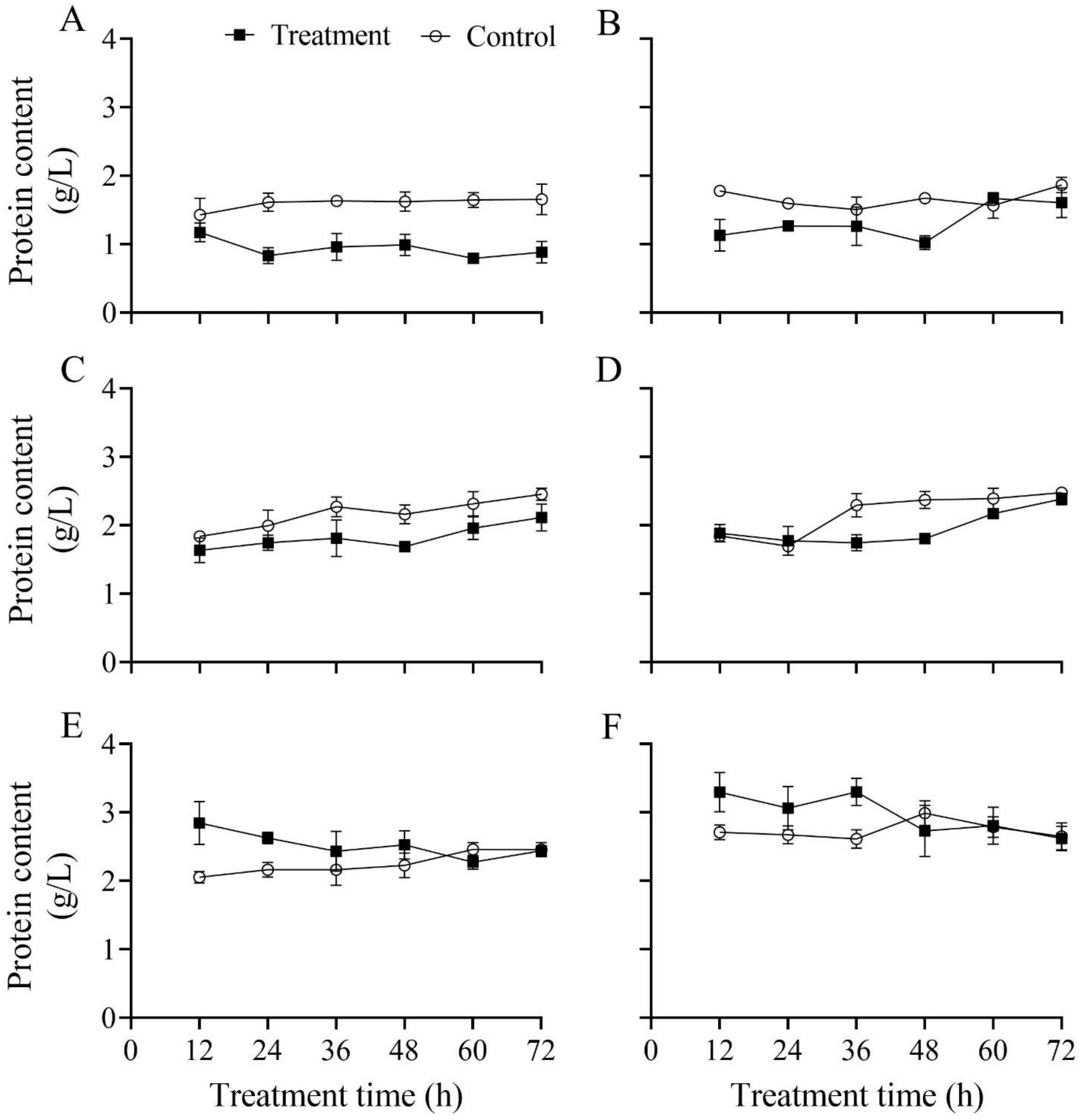

Figure S1. Effect of *B. bassiana* PfBb on larval protein content of *S. frugiperda*. A-F represents the first to sixth instar larvae, respectively.
